# Temperature-Specific Competition between Invasive Mosquitofish and an Endangered Cyprinodontid Fish

**DOI:** 10.1371/journal.pone.0054734

**Published:** 2013-01-23

**Authors:** Gerard Carmona-Catot, Kit Magellan, Emili García-Berthou

**Affiliations:** Institute of Aquatic Ecology, University of Girona, Girona, Catalonia, Spain; University of Sydney, Australia

## Abstract

Condition-specific competition is widespread in nature. Species inhabiting heterogeneous environments tend to differ in competitive abilities depending on environmental stressors. Interactions between these factors can allow coexistence of competing species, which may be particularly important between invasive and native species. Here, we examine the effects of temperature on competitive interactions between invasive mosquitofish, *Gambusia holbrooki*, and an endemic Iberian toothcarp, *Aphanius iberus*. We compare the tendency to approach heterospecifics and food capture rates between these two species, and examine differences between sexes and species in aggressive interactions, at three different temperatures (19, 24 and 29°C) in three laboratory experiments. Mosquitofish exhibit much more aggression than toothcarp. We show that mosquitofish have the capacity to competitively displace toothcarp through interference competition and this outcome is more likely at higher temperatures. We also show a reversal in the competitive hierarchy through reduced food capture rate by mosquitofish at lower temperatures and suggest that these two types of competition may act synergistically to deprive toothcarp of food at higher temperatures. Males of both species carry out more overtly aggressive acts than females, which is probably related to the marked sexual dimorphism and associated mating systems of these two species. Mosquitofish may thus impact heavily on toothcarp, and competition from mosquitofish, especially in warmer summer months, may lead to changes in abundance of the native species and displacement to non-preferred habitats. Globally increasing temperatures mean that highly invasive, warm-water mosquitofish may be able to colonize environments from which they are currently excluded through reduced physiological tolerance to low temperatures. Research into the effects of temperature on interactions between native and invasive species is thus of fundamental importance.

## Introduction

Condition-specific competition, a process by which competition between species is mediated by abiotic factors, is widespread in nature (e.g. [1], [2]). Species that inhabit spatially or temporally heterogeneous environments tend to have differing competitive abilities and varying tolerance for environmental stressors. Interactions between these factors can allow coexistence of competing species. In one scenario, for example, an inferior competitor may be excluded from part of its range, or for part of the time, by a competitively dominant species but be able to use other parts of its range, or more of its range at different times, through higher tolerance to an abiotic stress (e.g. [3], [4], [5]). In another scenario, a competitive reversal may occur whereby a competitively dominant species loses its advantage as conditions change along an environmental gradient and the previously subordinate species becomes dominant (e.g. [2], [6], [7]). Environmental gradients are particularly apparent in aquatic environments [2], [8], [9], which thus provide ideal situations in which to examine hypotheses concerning condition-specific competition. Several studies have investigated these phenomena in an array of taxa subject to various abiotic influences, including the effects of salinity on salt-marsh plants [6] and fish [1], hydroperiod on mosquitoes [7] and oysters [5], pH on amphibians [3], and temperature on stream fish [2], [4], [9].

Condition-specific competition may be particularly important when considering invasive species [5], [7], [10] and the dependence of competitive interactions between native and exotic species on temperature is receiving increasing interest [11], [12], [13], [14]. Temperature is a key factor for poikilothermic organisms and in freshwater and estuarine ecosystems temperature is often considered to be one of the dominant abiotic factors regulating interspecific competition [14], [15]. Moreover, growing concern regarding globally increasing temperatures means that research into the effects of temperature is of fundamental importance. In the Mediterranean region, for example, climate change models predict higher annual temperatures and longer droughts [16]. Interannual fluctuations are also expected to be more common, which would result in more exceptionally high temperature events [16]. In addition, continuing habitat alterations may lead to further increases in stream temperatures (e.g. [17]). These factors combined are likely to contribute to an expansion in range and population size of introduced warmwater fishes, and therefore increase predation rates or competitive effects on native species with preferences for cool water [18].

The eastern mosquitofish, *Gambusia holbrooki*, is a warmwater poeciliid fish native to the United States [19]. Since its introduction to Europe in 1921, *G. holbrooki* has established stable populations in most Mediterranean countries [20], [21]. However, *G. holbrooki* has not invaded northern Europe, probably because of reduced physiological tolerance, and therefore decreased competitive advantage, at lower temperatures (e.g. [20], [22]). Competition from mosquitofish has likely caused the displacement of several Mediterranean fish species, in particular cyprinodontiforms, from much of their native range [23], [24], [25]. For example, the Iberian toothcarp (*Aphanius iberus*), a cyprinodontid fish endemic to the Iberian Peninsula, originally occupied most of the Alt Empordà wetlands (NE Spain). Now only isolated populations remain while most of the coastal lagoons, ditches and rivers are inhabited by introduced mosquitofish [26]. From the original 38 Mediterranean populations, 15 are extinct [27] and the toothcarp is listed as Endangered (EN A2ce) under the IUCN Red List, and protected by a number of legislative frameworks such as the Bern Convention on the Conservation of European Wildlife and Natural Habitats [28].

The objective of this study is to examine the role of water temperature in determining the outcome of interspecific competition between invasive mosquitofish and native toothcarp. As mosquitofish are known to be aggressive [19], we predicted that they would exhibit both greater aggression and initiate more encounters, and that they would restrict toothcarp’s access to food. However, as mosquitofish are a warmwater species [19] we further predicted that any competitive advantage would be more evident at warmer temperatures, while at lower temperatures toothcarp would be able to benefit from *G. holbrooki*’s reduced competitive ability, thus demonstrating condition-specific competition. Finally, as both of these species show marked sexual dimorphism [19], [29] and males are generally more aggressive intraspecifically [30] but not always interspecifically (e.g. [31]), we expected sexual differences in aggressiveness.

## Methods

### Ethics Statement

All work was performed in compliance with Spanish laws of animal care and experimentation. The experiments were reviewed and approved by the Ethics Committee of the University of Girona.

### General Methods

Fish used in our experiment were captured using dip nets in September 2011 with scientific permits issued by the relevant authority (Generalitat de Catalunya, Direcció General del Medi Natural i Biodiversitat). Adult mosquitofish came from the Ter, Fluvià and Muga rivers near Girona, Spain, and toothcarp from Fra Ramon lagoon, Baix Empordà salt marshes, Spain [19]. About 200 fish of each species were transported to the laboratory and evenly distributed without mixing species in twelve 60 L species-specific stock aquaria (61 × 31 × 33 cm) containing a gravel substrate, conditioned water, and a filtered air supply. Mosquitofish from all three rivers were housed together. Aquaria were illuminated with 6 W bulbs and maintained at a constant photoperiod (12∶12 h light:dark cycle). The temperature was maintained at 24°C and fish were fed to satiation twice daily with commercial food flakes and frozen bloodworms (*Chironomus* spp.). Fish were allowed to adapt to laboratory conditions for at least four weeks prior to the start of temperature acclimation.

The temperature acclimation protocol was conducted in the same 12 aquaria, two for each species at each temperature, and consisted of the progressive adjustment of temperature using aquarium heaters until the three experimental temperatures (19, 24 and 29, ±0.2°C) were reached. These temperatures were selected because they are typical of the range of midsummer water temperatures found in Iberian coastal lagoons (e.g. [32]). Temperature was measured using digital thermometers placed inside the aquaria. After five days, all fish were at the necessary experimental temperature and were maintained at these conditions for at least 14 days before the start of observations. Mortality during acclimation was low (less than 5%) and only one fish died during observations. This trial was restarted after the fish was replaced. Fish acclimated to a specific temperature treatment were maintained at that temperature throughout the experimental period.

Observations were conducted in three 26 L aquaria (45 × 28 × 22 cm) also maintained at 19, 24 and 29°C respectively. Aquaria contained 2 cm of gravel substrate, were filled to a depth of 20 cm with conditioned water and were illuminated by 6 W lights. Dark plastic was attached to the back and sides of the aquaria to minimize disturbance. A removable, transparent methacrylate wall pierced with small holes (216 holes in 12 columns) divided each aquarium into two sides. During the afternoon before observations, fish were placed in the experimental aquaria at the same temperature as their respective acclimation temperatures. Two mosquitofish (visually size matched) of the same sex (50% of trials with males and 50% with females) were placed on one side of the methacrylate divider. Same sex mosquitofish were used to reduce the incentive for male-male competition over females. One toothcarp was randomly selected and its pair was then size matched; both fish were placed on the other side of the divider. The side for each species was swapped in successive trials. The methacrylate divider allowed the two species to visually and chemically respond to each other while preventing physical contact. Fish were fed to satiation with frozen bloodworms and uneaten prey were removed from the experimental aquaria. No food was provided to the experimental fish for at least 20 hours before observations. The series of experimental tests (i.e. Test 1, Test 2 and Test 3) were conducted sequentially the following day. To ensure that individual fish were used only once during the experiments, they were placed into post-experimental aquaria maintained at their specific acclimation temperature after the trials. Each of the three temperature treatments (19, 24 and 29°C) had 30 replicates (i.e. a total of 90 replicates with 360 different fish). All trials were videotaped (two sample videos at contrasting temperatures are provided in Movie S1 and Movie S2).

In test 1, we examined the tendency for mosquitofish and toothcarp to investigate and approach heterospecifics as a function of temperature. Observations began when the methacrylate divider was gently raised to the surface. Every care was taken to avoid disturbing the fish. We recorded the species and the time taken for the first fish to cross to the other side of the aquarium (specifically when the head or tail crossed the center line) and for the first fish to approach within one body length of the other species.

In test 2, following test 1, we studied the effects of temperature and sex on the agonistic interactions between mosquitofish and toothcarp. We waited five minutes after we raised the methacrylate divider to ensure that all fish were behaving normally and then conducted 10-minute observations recording the number of orientations (fish orienting itself and swimming towards another fish), nips (one fish attempts or succeeds at biting another) and chases (rapid chase of one fish by another). We conducted focal watches of one randomly selected fish per species sequentially, recording the sex of the fish observed for each species.

Test 3 immediately followed test 2. Here we assessed the effects of temperature on food competition between toothcarp and mosquitofish. Four bloodworms were placed at 10 cm intervals on a thin piece of wire and were carefully released at the water surface. Bloodworms were used because they are common prey items in the diet of the two species [33], [34]. We recorded the time taken to eat the first prey item and the species that consumed each of the four prey items. Any bloodworms that remained after five minutes were recorded as uneaten.

To assess the tendency for toothcarp and mosquitofish to investigate and approach conspecifics we used generalized linear models (GLMs) in a factorial design with two categorical factors, temperature and species. To analyze the proportion of each species over all trials for each temperature that were first to carry out these behaviors we used separate χ^2^ tests for each variable. For the agonistic variables we used separate GLMs for each species and each variable (orientations, nips and chases) with two categorical factors, temperature and sex. For the last experiment, we also used separate analyses for each species and GLMs for the proportion of prey eaten and the time taken to capture the first prey item with temperature as the single factor. In GLMs, we always used Poisson errors and log-link functions for count variables (i.e. number of nips, chases, and orientations), normal distributions and identity-link functions for time variables and binomial errors and logit-link functions for the proportion of prey eaten. Finally, we conducted two χ^2^ tests to assess the difference in the proportion of trials in which each species was the first to capture a prey item. First we included the uneaten prey items and second this category was excluded. All statistical analyses were conducted using the software SPSS 15.

## Results

**Figure 1:**
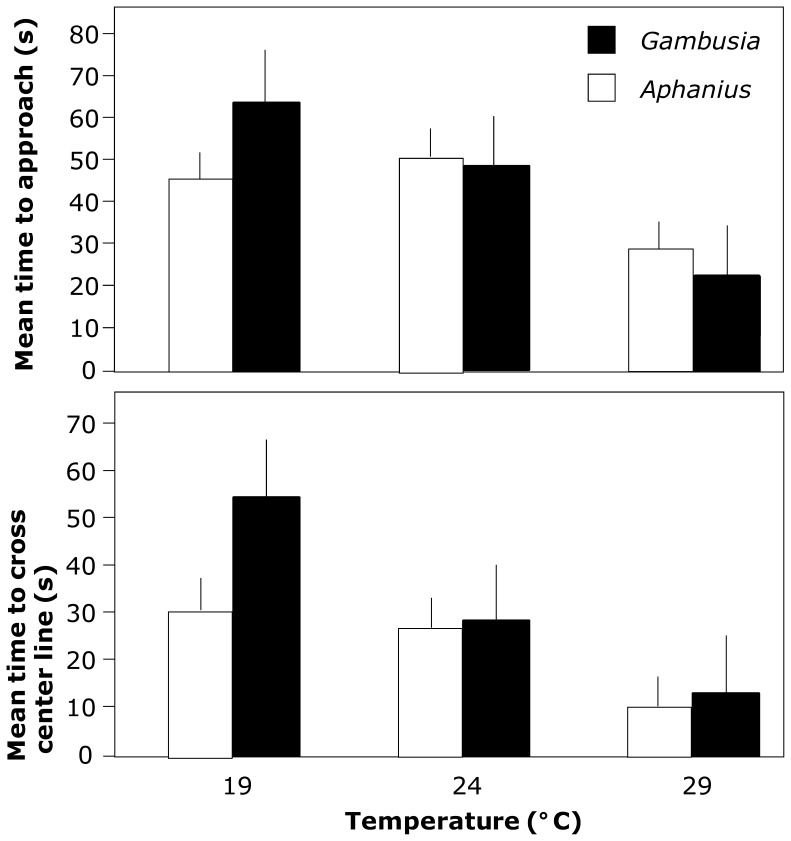
Mean time to a) cross the center line and b) approach a heterospecific for *G. holbrooki* and *A. iberus* as a function of temperature. Means and SE (error bar) are shown.

In test 1, the time taken for the first fish to cross the center line of the aquarium (GLM χ^2^ = 19.4, d.f. = 2, *P*<0.001) and the time taken for the first fish to approach within one body length of a heterospecific (GLM χ^2^ = 13.5, d.f. = 2, *P* = 0.001) both decreased significantly with increasing temperature (Figure 1). However, there was no difference between species in the time taken to carry out either of these behaviors (cross: GLM χ^2^ = 2.43, d.f. = 1, *P* = 0.119; approach: GLM χ^2^ = 0.086, d.f. = 1, *P* = 0.769), nor were the interactions significant (cross: GLM χ^2^ = 4.51, d.f. = 2, *P* = 0.105; approach: GLM χ^2^ = 4.86, d.f. = 2, *P* = 0.088). For the proportion of trials in which each species was the first to carry out these behaviors, toothcarp both crossed the center line first and approached a heterospecific first more often at 19°C, while this response was reversed at higher temperatures (cross: χ^2^ = 8.30, d.f. = 2, *P* = 0.016; 19°C, 22 toothcarp:8 mosquitofish, 24°C, 15∶15, 29°C, 12∶20; approach: χ^2^ = 7.23, d.f. = 2, *P* = 0.027; 19°C, 20∶10, 24°C, 11∶19, 29°C, 11∶19).

**Figure 2:**
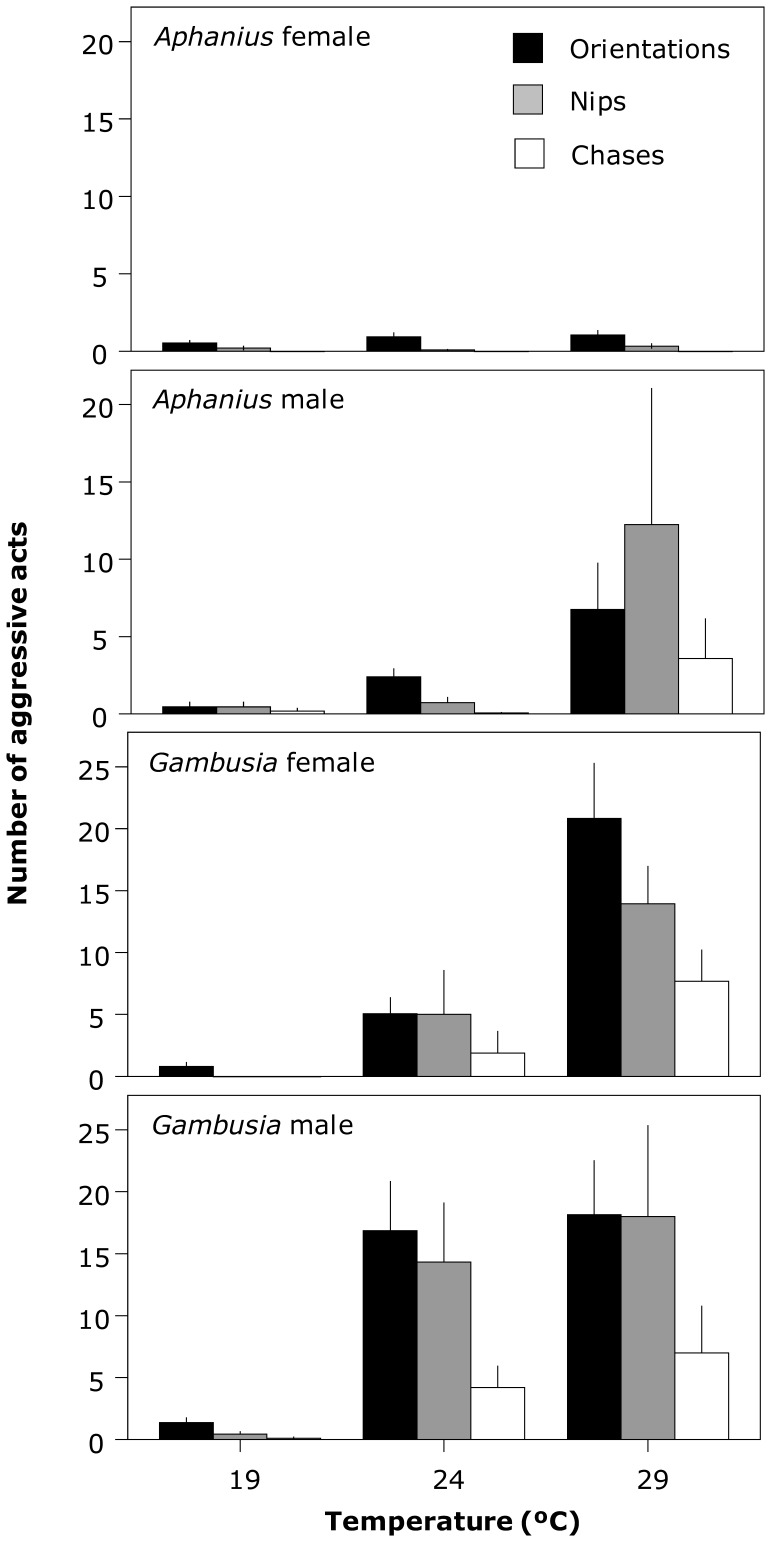
Aggressive acts (orientations, nips and chases) performed by *Gambusia holbrooki* towards *Aphanius iberus* and vice versa under the different temperature treatments and species combinations. Means and SE (error bar) are shown. Note the different scales on the *y*-axis for each species.

In test 2, mosquitofish exhibited much more aggression than toothcarp, with the majority of aggressive behavior being performed by mosquitofish towards toothcarp (84.02%), whereas only 15.98% was conducted by toothcarp towards mosquitofish. Aggression in both species varied significantly across temperatures (Table 1), with both species showing increased aggression with increasing temperature (Figure 2). There was also a significant difference between the sexes (Table 1). Males of both species exhibited more of all three of the recorded aggressive behaviors than females. Moreover, orientations appear to be the preferred behavior for females while males carried out relatively more nips to the extent that at the highest temperature the frequency of nips equaled or exceeded that of the other behaviors (Figure 2). Temperature × sex interactions were significant for almost all the behavioral variables (Table 1), with the exception of chases performed by toothcarp as female toothcarp did not carry out this behavior. Particularly, toothcarp males changed their preferred behavior type at 29°C from orientations to nips and particularly striking were the differing effects of temperature on male and female mosquitofish. Males exhibited the greatest increase in behaviors performed between 19 and 24°C while for females the major increase in behavior occurred at a higher temperature, between 24 and 29°C (Figure 2).

**Table 1:** Generalized linear models for selected response variables (i.e. orientations, nips, chases) of *Gambusia holbrooki* and *Aphanius iberus* using temperature and sex as predictors.

Species	Orientations	Nips	Chases
Source of variation			
*Gambusia*			
Temperature	300.29***	149.72***	82.31***
Sex	16.46***	64.81***	7.36 **
Temperature × Sex	75.33 ***	24.05***	11.70**
*Aphanius*			
Temperature	35.47***	31.56***	37.46***
Sex	16.46***	26.95***	–
Temperature × Sex	13.69**	11.88**	–

Values are χ ^2^.

“**”indicates *P*<0.01;

“***”indicates *P*<0.001; d.f. are 2 for temperature, 1 for sex, and 2 for their interaction.

**Figure 3:**
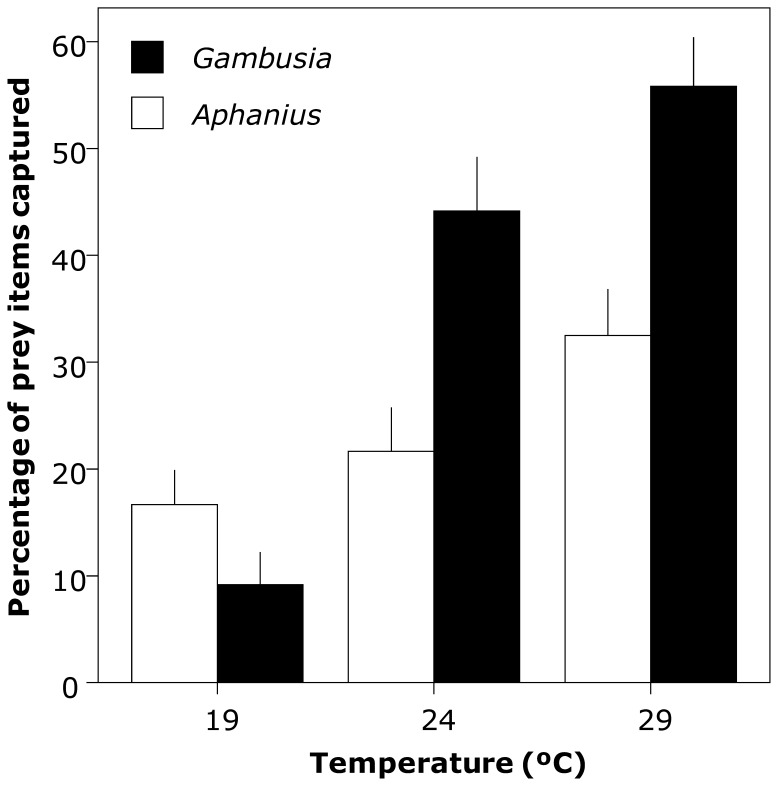
Effects of temperature on the number of prey items captured by *Gambusia holbrooki* and *Aphanius iberus*. Means and SE (error bar) are shown. Note that the totals for both species for 19 and 24°C do not equal 100%. This reflects the prey that remained uneaten.

In test 3 the proportion of prey items captured increased with temperature for both species. However, this relationship was significant only for mosquitofish (mosquitofish: GLM χ^2^ = 48.2, d.f. = 2, *P*<0.001; toothcarp: GLM χ^2^ = 3.05, d.f. = 2, *P* = 0.218; Figure 3). The time required to capture the first prey item decreased substantially between the lowest and highest temperatures (19°C: 29.9±57.1 s, 24°C: 56.2±83.4 s, 29°C: 8.6±15.4 s; mean ± s.d.) although this relationship was not straightforward and was only marginally significant (GLM χ^2^ = 5.92, d.f. = 2, *P* = 0.052). There was no significant difference between species (GLM χ^2^ = 3.41, d.f. = 1, *P* = 0.52; toothcarp: 19.0±36.9 s; mosquitofish: 42.8±76.5 s; mean ± s.d.) nor a significant interaction (GLM χ^2^ = 0.236, d.f. = 2, *P* = 0.89). When all trials at each temperature were considered together, at 19°C toothcarp captured the first prey item significantly more often than mosquitofish but this relationship was reversed for 24 and 29°C (χ^2^ = 25.2, d.f. = 4, *P*<0.001). However, this result was mainly due to the inclusion of uneaten prey items (toothcarp:mosquitofish:uneaten; 19°C, 13∶6:11; 24°C, 11∶18:1; 29°C, 13∶17:0). When this variable was removed the relationship between species and temperature was no longer significant (χ^2^ = 4.61, d.f. = 2, *P* = 0.11).

## Discussion

This study provides evidence for condition-specific competition, both through interference and exploitation, between eastern mosquitofish and Iberian toothcarp, via temperature-mediated changes in competitive abilities. Mosquitofish carried out close to five times as many aggressive acts as toothcarp, and while both species exhibited increased aggression at higher temperatures, this increase was considerably greater for mosquitofish than for toothcarp. Mosquitofish thus have the potential to competitively displace toothcarp through interference competition, and the strength of this interaction is likely to increase at higher water temperatures. Increased aggression at higher temperatures has been proposed as a major factor explaining the relative distribution of several fish species along longitudinal stream gradients [2], [4], [9]. For example, brook trout (*Salvelinus fontinalis*) were competitively dominant over cutthroat trout (*Oncorhynchus clarki*) at higher temperatures (20 versus 10°C), which was related to separation of these species into warmer, downstream (brook trout) and cooler, upstream (cutthroat trout) stretches of river [4]. In another study, brook trout were themselves subject to competitive exclusion by creek chub (*Semotilus atromaculatus*) at a slightly higher temperature (22°C) resulting in similar upstream-downstream species distributions [2]. In our study, temperature variation occurs more over a temporal rather than spatial scale, though microhabitat segregation of the two species through competitive interactions is also likely.

The time taken for the fish to both approach a heterospecific and to cross the center line of the experimental aquaria decreased with increasing temperatures although there were no differences between species. However, the frequency with which toothcarp were the first to cross the center line and approach a heterospecific was greater at 19°C, while at higher temperatures this situation was reversed and mosquitofish predominated. This seems to indicate a competitive reversal with toothcarp dominating at lower temperatures and mosquitofish superior at higher temperatures. However, the function of this approach behavior must be taken into consideration. The assumption that approaching a heterospecific is a prelude to attacking that individual is just one of a number of possibilities. For example, closer contact may be necessary to inspect a potential predator and hence assess the degree of threat [35], [36]. Alternatively, approaching other individuals may simply be a result of a predisposition for schooling behavior, as mixed species shoals are not uncommon [37], [38]. Whatever the purpose of this behavior, it is clear that toothcarp exhibited increased activity relative to mosquitofish at lower temperatures.

Temperature also influenced the potential for exploitative competition. The time taken to capture the first prey item decreased and the proportion of prey items captured by both species increased, with increasing temperature. However, there was no difference between species in capture time and the proportional increase in prey capture was significant only for mosquitofish. Furthermore, while toothcarp captured the first prey item more often at 19°C, this situation was reversed at 24 and 29°C, providing another example of potential competitive reversal. This situation relates to varying total food consumption by both species. Toothcarp captured the first food item with approximately the same frequency at all temperatures while mosquitofish increased their capture frequency at higher temperatures. Therefore, rather than toothcarp being more dominant at lower temperatures, they appear to benefit from reduced exploitative competition from mosquitofish. Release from dominance by a competitively superior species appears to be a common factor in competitive reversal. For example, in the brook trout – cutthroat trout system outlined above both species were nearly equal competitors at 10°C with brook trout becoming dominant only at the higher temperature [4]. A parallel pattern was shown in another study with the white-spotted char (*Salvelinus leucomaenis*) and the Dolly Varden char (*S. malma*) foraging equally well at lower temperatures but the former becoming dominant at a higher temperature [9]. A final consideration is that both these forms of competition, exploitative and interference, may be operating concurrently as in aggression to defend a food resource [39], [40]. Thus at higher temperatures mosquitofish have the capacity to restrict toothcarp access to food through exploitative competition and if food was limited, as is often the case, mosquitofish are likely to outcompete toothcarp through interference competition as well.

While aggression in the laboratory does not necessary imply competition in nature, in this case it is likely. Although interference competition is often more influential and clearer than exploitative competition [41], both types of competition can occur concurrently and interactively and may be difficult to distinguish [42]. *G. affinis* and *G. holbrooki* are well known to produce severe fin damage through nips, which can result in several adverse effects on recipient species [43], [44]. For example, swimming performance is likely to be reduced with potential consequent reduction in reproductive success and increased predation risk. Damage is costly in terms of regeneration effort and can increase susceptibility to disease [45], [46]. Fin damage can also result in changes in behaviour and prey consumption by the subordinate species [44], [47]. In the current study, the increase in aggression together with greater food capture efficiency shown by mosquitofish at higher temperatures indicates that mosquitofish have the capacity to outcompete toothcarp. Moreover, mosquitofish now dominate many of the habitats that were previously occupied by toothcarp [26] and competition is one of the likely mechanisms by which this has occurred.

There was a difference between males and females of both species in both the amount and type of behavior carried out, and for mosquitofish the temperature at which differences became apparent. Males of both species exhibited much more aggression than females. Moreover, females appear to prefer to engage in orientation behavior while males carry out more nips, particularly at higher temperatures, which is arguably a more aggressive behavior than merely observing another fish. These behavioral differences between sexes are likely to be associated with other differences. For example, in many animals, including fish, larger individuals initiate and receive less aggression [48], [49]. Both species in this study showed a marked sexual dimorphism with larger females and smaller males [19], [29] so this may account for some of the observed difference. In addition, females tend to be more sociable and engage in more shoaling than males [50], an activity incompatible with a high intensity of aggression. Finally, differences in aggression between the sexes may be an indirect consequence of the mating behaviors of these species [50]. Mating in mosquitofish is characterized by male coercion of females via sneaky mating, in which males attempt to insert their intromittent organ into the female’s genital opening by force and males compete aggressively for access to females [51], [52]. While reproductive behavior in toothcarp is less well studied, males do court females and will chase away rival males [53]. This may result in male predisposition for aggression [50], which is utilized to the detriment of heterospecifics. Because the temperatures used in this study were typical of the breeding season of both these species [54], [55], this effect may be intensified. Although male and female mosquitofish were not tested together in this study, behavior related to reproduction is likely to persist. An interesting result from this study is that male mosquitofish increased their level of aggression at 24°C, while females did not show a similar increase until 29°C. Males show a peak plateau in mating behavior in a comparable temperature range [56] though mating behavior was not quantified in our study. It also may be that males prefer cooler temperatures than females as is the case in two closely related species, *Poecilia sphenops* [57] and *Poecilia reticulata* [58]. Whatever the cause, for females their peak of maximum activity is either shifted to higher temperatures or is narrower compared to males, a factor which may influence the relative impact of males and females on toothcarp.

Temperature may have other effects that can interactively influence aggression. For example, the metabolic rate of ectotherms increases with increasing temperature (e.g. [59]), facilitating increased aggression. However, aggression itself is energetically costly [60] increasing metabolic rate still further, which probably accounts for the rise in food consumption at higher temperatures observed in this study. Another interacting factor is swimming speed which also increases with increasing temperature (e.g. [56]), which again will facilitate intensified aggression and again increase metabolic rate. In addition to these immediate effects, temperature variation may have long term consequences. In this study, fish were allowed to acclimate for four weeks. A longer duration of acclimation, can affect for example growth rate [47] and size at maturity [61]. Finally, temperature itself may interact with other factors, such as water velocity [62] and salinity [25] to influence aggressive activity.

We have shown here that temperature-specific competition may facilitate coexistence of native species with invasive mosquitofish. Mosquitofish have been introduced worldwide [61], [63] with far reaching effects on native species (reviewed in [64]) and are considered one of the 100 worst invasive species [65]. Therefore, any factor that may aid in ameliorating their effects should be investigated. The influence of temperature on interactions with mosquitofish has been examined in relation to several native species. For example, *G. holbrooki* aggression towards two Iberian toothcarp species (*A. iberus* and *Valencia hispanica*) increased at higher temperatures [23] and increased aggression with temperature has been shown by the closely related *G. affinis* towards *Galaxias maculatus* in New Zealand [43] and the least chub, *Iothichthys phlegethontis* in the USA [31], with effects on the survival of these native fish. In the current study, mosquitofish aggression may have immediate, medium and longer term consequences for toothcarp. In addition to disrupting normal conspecific interactions, mosquitofish can cause considerable fin damage [43], [44] and mortality, especially of juveniles [23], [43]. Injury, along with decreased food intake [24] and reduced growth rates [47] can lead to increased stress and susceptibility to illness [45], [46]. The temperatures used in this study are typical of breeding season temperatures for toothcarp, which is characterized by early offspring that can mature enough to breed later in the summer and late offspring that may overwinter and breed the following year [55]. Restriction of food and disruption of conspecific interactions is likely to reduce the breeding success of early offspring and overwinter survival of poor condition, late offspring could also be reduced. This in turn could result in changes in population demographics (e.g. [31]) through a decline in population density or a shift in breeding season and to the displacement of native species to non-preferred habitats (e.g. [43]). Climate change implies that investigating these types of temperature-mediated interactions between invasive and native species will be increasingly critical to aid in conservation efforts.

## Supporting Information

Movie S1 Movie showing an experimental trial at 29°C.
(MP4)

Movie S2 Movie showing an experimental trial at 19°C.
(MP4)
